# Predicting the risk for corneal graft rejection by aqueous humor analysis

**Published:** 2011-04-25

**Authors:** Philip Maier, Ulrike Heizmann, Daniel Böhringer, Yvonne Kern, Thomas Reinhard

**Affiliations:** University Eye Hospital, Freiburg, Germany

## Abstract

**Purpose:**

Cytokine patterns determined in the aqueous humor before penetrating keratoplasty (PK) may enable us to predict immune reactions (IR). We therefore analyzed 6 cytokines in the aqueous humor of patients before PK. By prospective clinical follow-up, we tested whether patients who developed an IR would present different preoperative cytokine patterns compared to patients without IR.

**Methods:**

We analyzed 18 samples of aqueous humor from 18 patients undergoing PK. The following cytokines were analyzed by cytometric bead array: interleukin 2 (IL-2), interleukin 4 (IL-4), interleukin 5 (IL-5), interleukin 10 (IL-10), tumor-necrosis-factor α (TNF-α), and interferon γ (INF-γ). Seven patients presented with signs of IR during follow up. We performed Cox proportional hazards analysis to determine significant predictors for IR. We iteratively eliminated all co-variates with p values over 0.1 from the survival model (backward selection).

**Results:**

Our final Cox model included the hazardous factors IL-4 (p=0.043) and INF-γ (p=0.059), protective factors IL-2 (p=0.081), IL-5 (p=0.028), and age at time of surgery (p=0.029). We performed a linear discriminant analysis based on these coefficients. The resulting function was: (−9.979*IL5) + (9.262*IL4) + (−3.928*IL2) + (1.709*IFN-γ) + (−0.183*age). A median of −4.97 separated patients with and without IR with no classification error.

**Conclusions:**

We demonstrate that cytokine levels in the aqueous humor can be predictive for IR. Our method allowed an almost 100% separation between patients with and without IR. This finding has the potential to improve the aftercare of PK fundamentally. However, our results need to be confirmed in a larger prospective cohort.

## Introduction

Corneal graft rejection remains the most important reason for graft failure [1]. Despite intense research, the precise mechanisms leading to graft rejection are not fully understood. Regarding the immunosuppressive climate in the anterior chamber, the contents of specific cytokines may correlate with the development of immune reactions or long-term clear graft survival. We have observed that active [2] but not total [3] transforming growth factor beta 2 (TGF-β_2_) is reduced during endothelial immune reactions. Moreover, we found that active TGF-β_2_ is increased in the aqueous humor of keratoconus patients before penetrating keratoplasty (PK), which might be one reason for their excellent graft prognosis [4]. Regarding the occurrence of immune reactions T helper cell type 1 (Th1)-type cytokines such as interleukin-2 (IL-2), interferon gamma (INF-γ), or tumor necrosis factor alpha (TNF-α) are more likely to be associated with allograft rejection than Th2-type cytokines such as interleukin-4 (IL-4), interleukin-5 (IL-5), interleukin-6 (IL-6), or interleukin-10 (IL-10) [5]. However, the exact effects of most cytokines in the aqueous humor regarding the development, maintenance and resolution of the endothelial immune reaction are not fully understood.

We therefore analyzed 6 different cytokines (IL-2, IL-4, IL-5, IL-10, INF-γ, and TNF-α) in the aqueous humor of patients before penetrating keratoplasty, following them prospectively. We compared the cytokine profiles of patients at the time of transplant in whom signs of endothelial rejection were or were not later found during the follow-up period.

## Methods

### Patients

All patients underwent penetrating keratoplasty for the first time at the University Eye Hospital Freiburg. None of the patients received topical steroids preoperatively. All invasive procedures were performed in adherence to the Declaration of Helsinki for research involving human subjects. Research was approved by our local ethics committee. After obtaining written informed consent, samples were drawn from a consecutive series of patients undergoing penetrating keratoplasty. Detailed clinical information on all study patients is summarized in Table 1.

**Table 1:** Graft and patient data of all study patients.

**Patient number**	**Patient age (years)**	**Gender**	**Indication**	**Risk profile**	**Postoperative treatment**	**Follow up (days)**	**IR**
patient 1	63	f	FT	N	S	1320	no
patient 2	80	f	B	N	S	487	no
patient 3	21	m	K	N	S	1084	no
patient 4	20	m	K	N	S	594	no
patient 5	49	m	HSV	H	M	639	no
patient 6	30	m	K	N	S	261	yes
patient 7	88	f	U	H	S	554	no
patient 8	79	f	FT	N	S	219	yes
patient 9	80	f	B	N	S	49	no
patient 10	47	f	K	N	S	173	yes
patient 11	62	m	U	H	M	519	no
patient 12	62	f	F	N	S	538	no
patient 13	73	m	HSVT	H	M	235	yes
patient 14	71	m	F	N	S	342	yes
patient 15	84	f	U	H	M	142	no
patient 16	41	m	F	N	S	441	yes
patient 17	56	f	F	N	S	730	no
patient 18	77	m	F	N	S	213	yes
mean/% all	60.1	50%f	na	72%N	78%S	474	na
mean/% without IR	60.4	64%f	na	64%N	73%S	605	na
mean/% with IR	60.7	29%f	na	86%N	86%S	269	na

PK=penetrating keratoplasty, ECD=endothelial cell density, IR=immune reaction. Gender: f=female, m=male. Indications: B=bullous keratopathy, F=Fuchs’ endothelial dystrophy, K=keratoconus, U=acute corneal ulcer, HSV=herpes simplex virus keratitis, T=triple procedure means combined surgery of penetrating keratoplasty with cataract extraction and posterior chamber lens. Risk profile: n=normal risk, H=high risk (definition see section patients and methods). Postoperative treatment: S=standard as described in the methods section, M=mycophenolate mofetil for 6 months postoperatively. ND=not determined, na=not applicable. For patients with immune reactions the time of follow-up reflects the time point of the occurrence of an immune reaction.

All keratoplasties were performed using mechanical trephines with a diameter of 8 mm. To fix the grafts, we used a double running cross-stitch suture with 10.0 nylon [6]. Gentamycine ointment was administered following surgery at least until the graft had been covered by a complete epithelial layer. Prednisolone-21-acetate 1% eye drops were then given 5 times daily and tapered during the first 5 postoperative months. For all patients included in the study systemic corticosteroids were administered for only 3 weeks postoperatively to protect the graft during the first post surgical period. Oral acetazolamide was administered at a daily dose of 2×250 mg for 5 days postoperatively. High-risk cases were defined by an increased risk for immune reactions as described elsewhere [7]. These patients’ postoperative treatment additionally included mycophenolate mofetil for 6 months postoperatively (see Table 1).

All grafts were preserved in organ culture according to the guidelines of the European Eye Bank Association. There were no significant differences for donor age, postmortem time, storage time and preoperative endothelial cell density between patients with and without graft rejection (data not shown).

Anterior chamber puncture was performed as described elsewhere  [8]. Briefly, a paracentesis lancet was used to penetrate the cornea in an avascular peripheral area over a length of 1 mm. Contact with limbal or peripheral corneal vessels was completely avoided. Aqueous humor (0.05-0.1 ml) was drawn into conventional tuberculin syringes without coming into contact with intraocular structures. All samples were rapidly frozen to −20 °C and kept at −80 °C until determination using cytometric bead array. To receive a representative and comparable sample size of patients with and without immune reaction some of the samples were drawn at the time of surgery and frozen afterwards. When the patients later during follow up presented with an endothelial immune reaction, the samples were thawed and the cytokine analysis was performed. By this we artificially increased the number of patients with immune reactions, so that in the normal risk group the ratio of patients with immune reaction was higher than in the high risk group. Therefore, the number of patients with immune reactions in the normal risk group is higher than it would be expected.

Only endothelial immune reactions were included as the primary endpoint in this study and were diagnosed at the slitlamp when endothelial immune precipitates accompanied by stromal edema were visible.

The cytokine levels in each sample were measured using a Cytometric Bead Array (CBA kit; RD Bioscience, San Diego, CA) according to the manufacturer's manual and described elsewhere [9]. Data were acquired by flow cytometry (FACS Clibur; BD Bioscience). For our analyses, we used the Th-1/Th-2 Kit (RD Bioscience, San Diego, CA) for determining IL-2, IL-4, IL-5, IL-10, TNF-α, and INF-γ. All values were calculated with regards to the negative control in each assay. If the fluorescent signal of a sample was equal to or below the fluorescence of the negative control, the cytokine level was set as 0.0 pg/ml.

All statistical computations were performed using the R-Software system [10]. To achieve better comparability between the different cytokines we normalized all data by dividing each cytokine value by its respective maximum in the whole cohort before analysis, so that all cytokine values were between 0 and 1. We performed Cox proportional hazards analysis to determine the significant predictors for IR during follow up. We entered age at time of surgery and the normalized cytokine levels as co-variates into the model. We iteratively eliminated all co-variates with p values higher than 0.1 from the survival model by backward selection. The final model consisted of co-variates with p-values less than 0.1.

## Results

All of each patient’s cytokine levels and the respective cytokine scores are summarized in Table 2. For patients without immune reaction levels of IL-2 (mean 1.7 versus 1.1 pg/ml) and IL-5 (mean 1.6 versus 0.9 pg/ml) as well as age (mean 60.4 versus 59.7 years) were slightly higher and levels of IL-4 (mean 1.8 versus 2.5 pg/ml), IL-10 (mean 1.2 versus 1.7 pg/ml), TNF-α (1.2 versus 1.7 pg/ml), and INF-γ (1.0 versus 1.1 pg/ml) were slightly lower than for patients with immune reaction. In an earlier study [11] we determined the same cytokines in control patients (n=26) receiving cataract surgery only and found the following cytokine levels that might be regarded as normal values (mean±standard deviation): 1.64±0.91 pg/ml for IL-2, 1.86±1.13 pg/ml for IL-4, 1.16±0.50 pg/ml for IL-5, 1.51±1.17 g/ml for IL-10, 1.23±0.76 pg/ml for TNF-α, and 0.62±1.18 pg/ml for INF-γ. Regarding the absolute levels of each cytokine all values fell within the range of the normal levels. However, we wanted to test the hypothesis that it as a complex network of cytokines that may be responsible for the individual rejection risk of the patients. Therefore, we did not compare the cytokine levels for each individual cytokine but performed the Cox proportional hazards analysis to find a significant combination of parameters to differentiate between patients with and without graft rejection.

**Table 2:** Detailed information on cytokine levels.

	**Cytokine**								
**Patient number**	**Il-2 (pg/ml)**	**IL-4 (pg/ml)**	**IL-5 (pg/ml)**	**IL-10 (pg/ml)**	**INF-γ (pg/ml)**	**TNF-α (pg/ml)**	**Age (years)**	Cytokine score*	**IR**
patient 1	1.2	1.4	1.1	1.2	0.0	0.0	63	−14.20	no
patient 2	1.5	1.7	1.3	2.5	0.0	1.4	80	−17.75	no
patient 3	1.9	1.9	1.2	1.7	0.0	1.5	21	−5.63	no
patient 4	1.8	0.0	1.5	1.5	1.8	1.2	20	−22.56	no
patient 5	1.5	0.0	1.2	1.2	2.8	1.4	49	−22.07	no
patient 7	2.3	3.7	2.3	4.3	4.7	0.0	88	−5.85	no
patient 9	1.2	1.4	0.0	1.6	1.2	0.0	80	−4.31	no
patient 11	1.2	1.8	2.0	4.0	0.0	0.0	62	−19.26	no
patient 12	2.2	3.1	1.2	1.5	0.0	1.6	62	−3.29	no
patient 15	0.0	1.7	3.9	2.3	2.2	1.2	84	−34.85	no
patient 17	4.0	3.2	1.7	2.1	0.0	2.4	56	−13.25	no
patient 6	0.0	3.6	2.9	0.0	0.0	4.5	30	−1.02	yes
patient 8	0.0	1.8	0.0	1.4	0.0	0.0	79	+2.25	yes
patient 10	0.0	1.2	0.0	0.0	0.0	0.0	47	+2.56	yes
patient 13	2.1	1.3	1.0	29.1	11.6	0.0	73	+0.24	yes
patient 14	3.8	5.7	2.2	3.9	0.0	3.0	71	+2.87	yes
patient 16	1.5	2.0	0.0	0.0	0.0	0.0	41	+5.06	yes
patient 18	0.0	1.7	0.0	0.0	0.0	0.0	77	+1.74	yes
mean/all	1.5	2.1	1.3	3.2	1.4	1.0	60.1	−8.30	na
median/all	1.5	1.75	1.2	1.6	0.0	0.6	62.5	−4.97	
mean/without IR	1.7	1.8	1.6	2.2	1.2	1.0	60.4	−14.82	na
median/without IR	1.5	1.7	1.3	1.7	0.0	1.2	62.2	−14.20	
mean/with IR	1.1	2.5	0.9	4.9	1.7	1.1	59.7	+2.0	na
median/with IR	0.0	1.8	0.0	0.0	0.0	0.0	71.3	+2.25	

IR=immune reaction, na=not applicable. *The cytokine score has been calculated as described above including the regression coefficients from the Cox model.

When we tested whether the underlying disease leading to penetrating keratoplasty also influenced the cytokine levels in the aqueous humor of the different patients, we found a statistically significant difference between the indication groups (bulluos keratopathy (n=1), Fuchs endotehlial dystrophy (n=8), keratoconus (n=4), herpetic eye disease (n=2), corneal ulcers (n=3)) regarding age at time of surgery (p=0,01, *t*-test), and the INF-γ level (p=0,003, *t*-test) in the aqueous humor. Regarding age we found the youngest patients as expected in the keratoconus group. For INF-γ we found highest levels in the eyes with herpetic eye disease. However, these were only two cases where one developed an immune reaction and the other did not.

**Figure 1:**
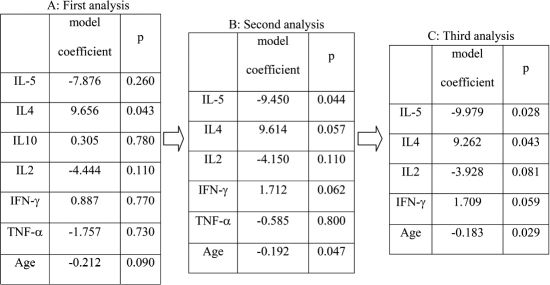
Backward selection. As IL10 showed the least significant result (p=0.78) in the first analysis (**A**), it was excluded from the second analysis. In the second analysis (**B**), TNF-α showed the least significant result (p=0.80), and was excluded from the third analysis (**C**). As the level of significance for backward selection was chosen to be p<0.1, all factors included in analysis three (age, IL2, IL4, IL5, and IFN-γ) were included in the final Kaplan–Meier survival analysis.

Backward selection of the Cox proportional hazards model is summarized in Figure 1. The third and final analysis for predicting immune reactions revealed IL-2, IL-5, and age to have significant graft protecting effects and IL-4 and INF-γ to be significant hazardous factors.

Regarding the model coefficients of each factor, IL-5 (model coefficient: −9.979, hazard ratio: 0.00046), IL2 (model coefficient: −3.928, hazard ratio: 0.02) and age (model coefficient: −0.183 per year, hazard ratio: 0.83) demonstrated graft protecting effects in which IL-5 was the strongest factor preventing immune reactions. On the other hand, IL-4 (model coefficient: +9.262, hazard ratio: 10500) and INF-γ (model coefficient: +1.709, hazard ratio: 5.52) revealed effects promoting the development of immune reactions, while IL-4’s effect seemed to be stronger than that of INF-γ. We derived a linear discriminant function (Equation 1) on the basis of the coefficients from our final Cox model. We evaluated the score from this function for differentiating between patients with and those without immune reactions in a second step [(Equation 1: f(age, IL5, IL4, IL2, IFN)=(−9.979*IL5) + (9.262*IL4) + (−3.928*IL2) + (1.709*IFN-γ) + (−0.183*age)].

**Figure 2:**
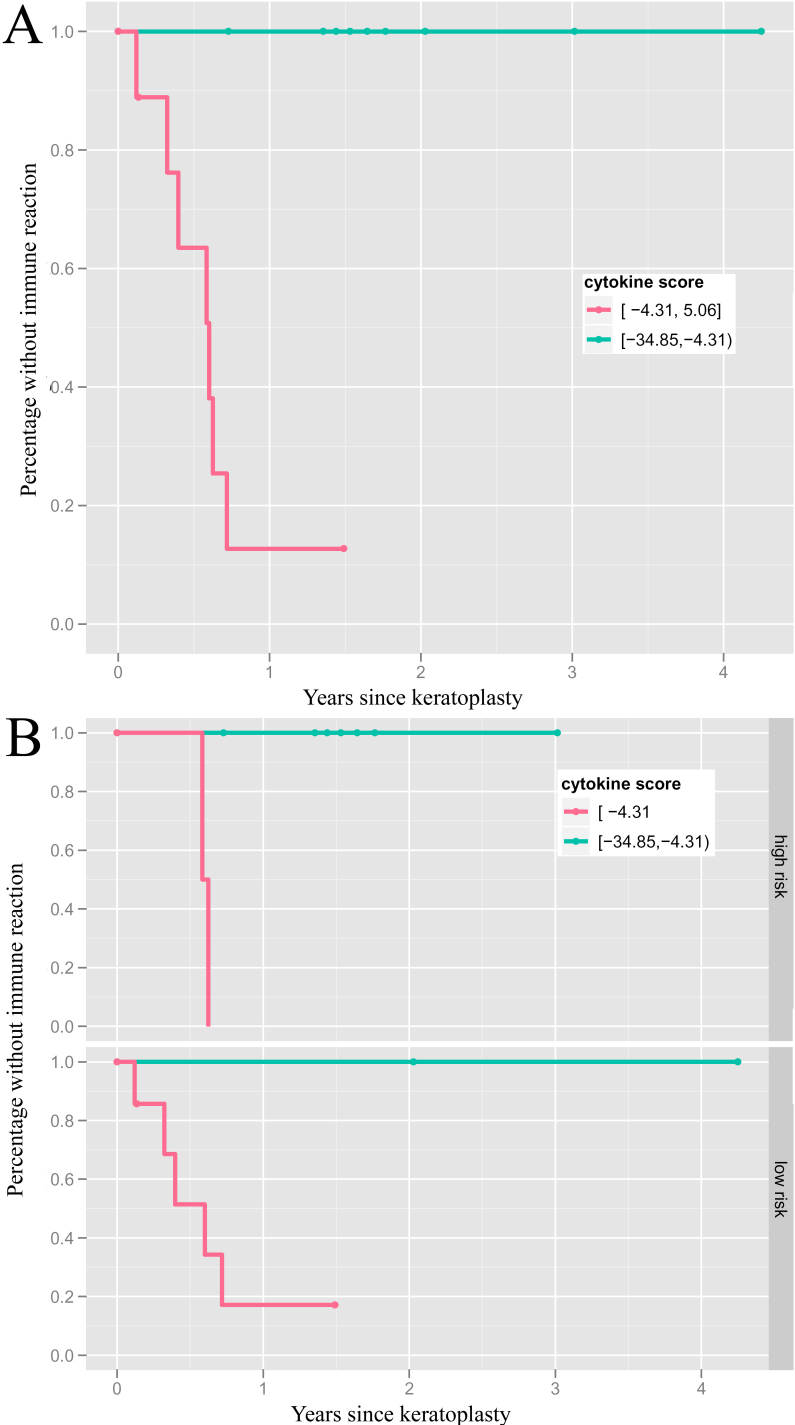
Occurence of immune reactions separated by the median of the cytokine score. Kaplan–Meier survival analysis showing an almost 100% separation accuracy between patients with and without immune reactions following PK by the median (−4.97) of the calculated cytokine score (**A**, n=9 for each group). This differentiation is independent from the patients’ risk profile (**B**, definition for high risk situations see section patients and methods).

The cytokine score’s median in the whole study group was −4.97, ranging from: −34.85 to +5.06. The median separated precisely the patients with and those without IR (see Figure 1 and Figure 2A). There was no difference between high and normal risk patients in the accuracy of classification (Figure 2B), so that the use of mycophenolate mofetil did not seem to bias the prediction value of the cytokine score.

## Discussion

This is the first study to correlate prospectively determined cytokine levels in the aqueous humor of patients before PK to the occurrence of endothelial immune reactions during the postoperative course. Our statistical analyses revealed IL-2, IL-4, IL-5, INF-γ, and age at time of surgery as most predictive for immune reactions after PK. A simple linear discriminant function based on these five factors resulted in an almost 100% accurate prediction.

However, the distinct cytokine profile may merely reflect a breakdown of the ocular immune privilege in the high-risk cases. If so, the clinical high-risk rating would be equally predictive of immune reactions. Anyhow, the cytokine-score retained predictive power in a Cox model that also included the high- versus low-risk factor (not shown). This fact emphasizes, that the cytokine profile adds predictive information to the clinical rating into low- versus high-risk.

Although the absolute cytokine levels for all patients reported in this study fell in the range of normal values that were determined in an earlier study, it was the combination of the different cytokine levels resulting from the Cox proportional hazards analysis that allowed a differentiation between patients with and without rejection. This confirms the hypothesis, that it is not a single cytokine that is responsible for an intact immune privilege but a complex network of various cytokines, so that immune reactions occur only if a combination of parameters within this network are changed simultaneously. The results of this study may therefore help to understand which cytokines are more or less important in the context of endothelial immune reactions following corneal transplantation.

Besides the 6 cytokines included in this study there are many more cytokines that play an important role in the development, the maintenance and the resolution of immune reactions (e.g., TGF-β2, IL-6, and α-MSH). However, due to a limited sample volume we had to choose for one multiplex bead array that included only the 6 cytokines reported in this study.

Regarding the underlying disease leading to penetrating keratoplasty that might also influence the cytokine levels in the aqueous humor of the patients we found only statistically significant differences for INF-γ which levels were highest in patients with herpetic eye disease. As these were only two cases of which one developed an immune reaction and the other one did not, we think that there seems to be no significant impact of the underlying disease on the overall cytokine score used to differentiate between patients with and without immune reaction.

### Graft protecting factors

The strongest graft protecting factor in this study was IL-5, which stimulates B-cell growth, and promotes the immature phenotype of antigen-presenting cells. In an animal model of heart transplantation, IL-5 was shown to potentially prolong allograft survival by down-regulating IL-2 and INF-γ production [12]. In animal experiments, antigen presenting cells from the central cornea have been shown to migrate into the recipient’s lymphatics giving rise to allorecognition and graft rejection [13]. As IL-5 is capable of promoting the immature phenotype of antigen presenting cells in vitro, it may have graft protecting effects in a clinical setting [14].

We found that the pro-inflammatory, Th1-related cytokine IL-2 revealed graft-protecting rather than IR-promoting effects in the aqueous humor of patients. As IL-2 is believed to be a barrier to tolerance, thus leading to immune reactions [15], one would have anticipated that high levels of IL-2 would increase the risk of immune reaction. However, there is evidence that the survival times of IL-2 knockout mice were only modestly reduced. Thus IL-2 does not seem necessary for allograft rejection [16] and the potential immune reaction-inducing effect of IL-2 on T-cell proliferation may be strong in vitro, but somehow paradoxical in vivo following PK.

### Factors increasing the risk for immune reactions

As IL-4 is an anti-inflammatory cytokine secreted by activated T-cells it was surprising that it was the strongest factor increasing the risk for an endothelial immune reaction. Graft survival has been successfully prolonged by the in vivo administration of IL-4 used in experimental models of solid organ transplantation [17]. However, IL-4 overexpression was not sufficient to reduce the rejection rate of corneal allografts in a rat keratoplasty model using a gene therapy approach [18]. As IL-4, unlike IL-5, fosters the differentiation of antigen presenting cells in vitro [14], IL-4 might contribute to graft rejections by inducing the differentiation of the graft’s immature myeloid antigen-presenting cells. These contradictory results reveal that IL-4 may exert both graft protecting and immune reaction promoting effects in organ transplantation [15].

As INF-γ is a strong pro inflammatory cytokine that strengthens the Th-1 immune response, it is not surprising that increased levels in the aqueous humor lead to a higher risk of endothelial immune reactions following PK. We also found that INF-γ is statistically significantly increased [11], whereas TGF-β_2_, the counterpart of INF-γ, is statistically significantly decreased in the aqueous humor of patients during an active immune reaction [2].

### Cytokines excluded from the final Cox model

TNF-α is believed to be important in the initiation, maintenance, and resolution of inflammation regarding inflammatory processes leading to graft rejection. In a mouse model of corneal transplantation, Zhu et al. [19] showed that TNF-α expression generally decreases during the first postoperative week and remains significantly elevated in allogeneic (but not in syngeneic) grafts, implicating TNF-α as a mediator of the alloimmune response in corneal transplantation. Perhaps this explains why we did not find TNF-α to be prognostically significant regarding the occurrence of immune reactions when determined before penetrating keratoplasty. Its importance may lie in the maintenance, not the induction, of immune reactions.

Although IL-10 is considered one of the most promising immunosuppressive cytokine candidates, exogenous IL-10 administration did not prolong corneal graft survival in a rat model of allotransplantation [20]. Animals injected subconjunctivally with IL-10 even showed a tendency toward earlier rejection when compared to controls [20]. Furthermore, we found statistically significantly increased IL-10 levels in the aqueous humor of patients having an active immune reaction compared to patients without immune reactions or controls [11]. Gong et al. [21] demonstrated that only systemic but not topical application of IL-10 gene vectors prolonged corneal graft survival in a rat keratoplasty model. They concluded that IL-10 modulates cytokine expression in the draining lymph nodes, leading to graft-protecting effects. We did not find that IL-10 is prognostically important in predicting the occurrence of immune reactions in this study. Therefore, as we know that cytokines can display paradoxical effects [22], further investigation is required to determine the role of IL-10 in corneal graft acceptance or rejection.

### Genetic polymorphism of cytokines and growth factors

Genetic cytokine and growth factor polymorphisms may be responsible for the variation of cytokine levels in the aqueous humor, since single nucleotide polymorphisms (SNPs) have been shown to influence protein secretion in vitro and in vivo. This allows to categorize individuals as high, low or intermediate producers of a given cytokine [23]. However, the role of cytokine SNPs in affecting the immune response to the allograft or drug therapy on corneal transplantation has not been investigated. Therefore, including SNP analysis may help to develop a more complex score system to predict immune reactions using a multi-variant approach based on the genetic profile, on cytokine levels in the aqueous humor and other relevant clinical risk factors.

The major limitation of this study is the fact that we have not yet confirmed our findings in a second, larger, prospective, and independent cohort. Nevertheless, we observed a cytokine score that allows us to distinguish with almost 100% accuracy between patients developing an endothelial immune reaction following PK from those who do not. Therefore, our cytokine score, including IL-2, IL-4, IL5, INF-γ, and age, may prove to be a helpful tool in predicting the risk of endothelial immune reaction at the time of surgery in the future. Patients with a high cytokine score might then be treated as high-risk patients, and thus undergo systemic immunosuppressive treatment to prevent the occurrence of immune reactions. However, our study results and the cytokine score need to be verified in a larger study population and the importance of those cytokine SNPs must also be examined before a modified score may be incorporated into clinical practice.
